# Role of Haptoglobin in Polycystic Ovary Syndrome (PCOS), Obesity and Disorders of Glucose Tolerance in Premenopausal Women

**DOI:** 10.1371/journal.pone.0005606

**Published:** 2009-05-19

**Authors:** Francisco Álvarez-Blasco, Ma Ángeles Martínez-García, Manuel Luque-Ramírez, Naiara Parraza, José L. San Millán, Héctor F. Escobar-Morreale

**Affiliations:** 1 Department of Endocrinology, Hospital Universitario Ramón y Cajal & Universidad de Alcalá, Madrid, Spain; 2 Centro de Investigación Biomédica en Red Diabetes y Enfermedades Metabólicas Asociadas CIBERDEM, Instituto de Salud Carlos III, Spanish Ministry of Science and Innovation, Madrid, Spain; 3 Department of Clinical Biochemistry, Hospital Universitario Ramón y Cajal, Madrid, Spain; 4 Department of Molecular Genetics, Hospital Universitario Ramón y Cajal, Madrid, Spain; National Institute of Child Health and Human Development/National Institutes of Health, United States of America

## Abstract

**Background:**

*Hp^2^* alleles of the haptoglobin α–chain polymorphism reduce the anti-oxidant properties and increase the pro-inflammatory actions of this acute-phase protein in a gene-dosage fashion. We hypothesized that the haptoglobin polymorphism might contribute to the increased oxidative stress and low-grade chronic inflammation frequently associated with polycystic ovary syndrome, obesity, and abnormalities of glucose tolerance.

**Methodology/Principal Findings:**

Serum haptoglobin and the haptoglobin α–chain polymorphism were determined in 141 patients with polycystic ovary syndrome and 102 non-hyperandrogenic women. Of the whole group of 243 premenopausal women, 117 were obese and 51 showed abnormal glucose tolerance. Although serum haptoglobin concentrations were similar in PCOS patients and controls, the former presented with an increased frequency of *Hp^2^* alleles (62% vs. 52%, *P* = 0.023). Circulating haptoglobin levels increased with obesity (*P*<0.001), yet no association was found between obesity and haptoglobin genotypes. No differences were observed in haptoglobin levels or genotype frequencies depending on glucose tolerance. Fifty percent of the variation in serum haptoglobin concentrations was explained by the variability in serum C-reactive protein concentrations, BMI, insulin sensitivity and haptoglobin genotypes.

**Conclusions/Significance:**

Serum haptoglobin concentrations in premenopausal women are largely dependent on the haptoglobin polymorphism and on the presence of obesity, with insulin resistance and chronic inflammation possibly modulating this relationship. The association of polycystic ovary syndrome with *Hp^2^* alleles suggests that the anti-oxidant and anti-inflammatory properties of haptoglobin may be reduced in these patients.

## Introduction

Polycystic ovary syndrome (PCOS), a very prevalent syndrome characterized by the association of hyperandrogenism with chronic ovulatory dysfunction in premenopausal women [1], [2], is among the most common metabolic associations of both type 1 [3], [4] and type 2 diabetes mellitus [5]. Hyperinsulinism and insulin resistance are frequent findings in PCOS patients, and these traits have cause-consequence relationships with obesity [6], increased serum ferritin concentrations [7], low-grade chronic inflammation [8], oxidative stress [9], and increased cardiovascular risk [10].

We have shown that serum ferritin concentrations are increased in PCOS patients independently of chronic inflammation, suggesting increased body iron stores in these women very especially when obesity is also present [7]. Furthermore, our recent results strongly suggest that insulin resistance and hyperinsulinism, and not the reduced menstrual losses derived from oligomenorrhea or mutations in the hemochromatosis *HFE* gene, underlie the origin of these increased body iron stores [11], [12].

But iron metabolism is a complex system in which protection against iron losses and against the oxidative damage caused by free iron radicals are predominant mechanisms [13]. Haptoglobin, a circulating acute-phase protein that is synthesized in the liver under the influence of several cytokines such as TNF-α and interleukin-6 [14], ameliorates the iron loss and renal injury that follows the liberation of hemoglobin from erythrocytes during processes of intravascular hemolysis. Haptoglobin accomplishes this function by forming soluble complexes with hemoglobin, thereby reducing the generation of oxygen reactive species and ameliorating oxidative stress [14]. Also, haptoglobin inhibits the synthesis of prostaglandins and the vasodilation mediated by nitric oxide, and exerts immunomodulatory actions by its binding to the CD163 receptor present in macrophages and monocytes [14].

The molecular structure of haptoglobin is determined in humans by the existence of a polymorphism affecting the gene on chromosome 16q22 that encodes the α chain of this globulin. This polymorphism results in the existence of two different alleles, termed *Hp^1^* and *Hp^2^* [15] that may be explored by DNA genotyping or by plasma gel electrophoresis phenotyping [14]. The *Hp^2^/Hp^2^* genotype = Hp 2-2 phenotype reduces plasma haptoglobin levels, decreases its hemoglobin-binding capacity and anti-oxidant properties and the secretion of anti-inflammatory cytokines such as IL-10, while increasing immunological reactivity [16].

Furthermore, haptoglobin phenotypes influence serum ferritin concentrations, and increased ferritin concentrations have been described not only in PCOS, but also in insulin resistant disorders such as obesity, type 2 diabetes mellitus and the metabolic syndrome [13]. Men expressing the Hp 2-2 phenotype present with increased serum ferritin concentrations when compared with men expressing the Hp 2-1 and Hp 1-1 phenotypes [17]. Haptoglobin is expressed in adipocytes [18] and, because serum haptoglobin concentrations correlate with increasing body weight, its circulating concentrations are increasingly being considered as a marker of adiposity. Finally, a recent proteomic analysis of serum has identified both the α and β haptoglobin chains as potential PCOS biomarkers [19], [20].

Therefore, we conducted the present study hypothesizing that haptoglobin and its polymorphism might mediate the associations of PCOS with hyperferritinemia and increased body iron stores, obesity and disorders of glucose tolerance.

## Materials and Methods

### Ethics statement

Written informed consent was obtained from all the participants and the study was approved by the Clinical Research Ethics Committee of Hospital Universitario Ramón y Cajal.

### Participants

We studied serum haptoglobin concentrations and haptoglobin genotypes in a large series of obese and non-obese PCOS patients and non-hyperandrogenic controls presenting with or without abnormalities of glucose tolerance. The sample included 141 PCOS patients and 102 non-hyperandrogenic Caucasian women prospectively recruited at the Department of Endocrinology of Hospital Universitario Ramón y Cajal, a tertiary care academic hospital affiliated with the University of Alcalá. The diagnosis of PCOS relied on the presence of oligo-ovulation and clinical and/or biochemical hyperandrogenism, after excluding specific etiologies [1], [2], [21]. The particular methods used to establish these criteria have been reported earlier [6]. The non-hyperandrogenic control group was composed of normal weight healthy female volunteers and of consecutive overweight or obese women, who did not have any known metabolic comorbidity, reporting to the clinical practice of the authors solely for treatment of weight excess. Controls were selected in order to be similar in terms of BMI with the patients. None of the controls had signs or symptoms of hyperandrogenism, menstrual dysfunction, or history of infertility.

According to their body mass index (BMI), women were classified as being obese (BMI≥30 kg/m^2^) or non-obese (BMI<30.0 kg/m^2^), because only obesity - and not overweight - has demonstrated a negative impact on mortality [22].

None of the participants had a personal history of hypertension, regular alcohol consumption, disorders of glucose tolerance, hyperuricemia, cardiovascular events, sleep apnea, or had received treatment with oral contraceptives, antiandrogens or insulin sensitizers for the previous 6 months.

### Study protocol and assays

Clinical and anthropometrical variables, including the hirsutism score, BMI and waist-to-hip ratio were determined as reported previously [6]. Blood samples were obtained between days five and 10 of the menstrual cycle, or during amenorrhea after excluding pregnancy, in all the participants. After a three-day 300-g carbohydrate diet and 12-h overnight fasting, samples were obtained early in the morning for the measurement of haptoglobin, ferritin, C-reactive protein, total testosterone, sex hormone-binding globulin and calculated free testosterone [23], androstenedione, estradiol, dehydroepiandrosterone-sulfate, 17-hydroxyprogesterone, prolactin, thyrotropin, luteinizing hormone and follicle-stimulating hormone. Complete serum biochemistry and lipid profiles were also obtained. Then, a 75-g oral glucose tolerance test was performed, and samples were obtained for measurement of serum insulin and plasma glucose at 0, 30, 60, 90 and 120 min. Samples were immediately centrifuged, and serum and plasma were separated and frozen at −30 C until assayed.

The circulating concentrations of haptoglobin were assayed by a commercial immunonephelometry method (Dade Behring, Marburg, Alemania) calibrated against the international CRM 470 reference material [24] with intraassay and interassay CV of 2.6% and 6.2%, respectively. Haptoglobin genotypes were estimated using the PCR-based technique previously described by Koch *et al.* [25]. For plasma haptoglobin levels, we used the normal ranges established internationally for each particular haptoglobin genotype/phenotype [14].

Serum ferritin and C-reactive protein concentrations were measured by automated immunochemiluminescence (Immulite 2000 Ferritin and High Sensitivity CRP, Diagnostic Products Corporation, Los Angeles, CA) with lower limit of detection of 0.88 pmol/l and 0.1 mg/l respectively, and intra- and inter-assay coefficients of variation below 10%.

The technical characteristics of the assays employed for plasma glucose concetrations, lipid profiles and serum hormone measurements have been described elsewhere [6]. The composite insulin sensitivity index was calculated from the circulating glucose and insulin concentrations during the oral glucose tolerance test according to Matsuda and DeFronzo [26], and women were classified as having normal or abnormal glucose tolerance (impaired fasting glucose, impaired glucose tolerance or diabetes) according to the American Diabetes Association clinical practice recommendations [27].

### Statistical analysis

The results are reported as mean±standard deviation unless otherwise stated. The association of the haptoglobin genomic variants with PCOS, obesity and abnormalities of glucose tolerance was explored using the χ^2^ or Fisher's exact test as appropriate. The Kolmogorov-Smirnov statistic was applied to continuous variables. Logarithmic or square root transformations were applied as needed to ensure normal distribution of the variables, and parametric tests were applied thereafter. Mathematical transformation was not applied to serum haptoglobin levels, because this variable was normally distributed.

General linear models adjusting for age served to evaluate the impact of PCOS on clinical and biochemical variables. General linear models introducing the haptoglobin genotype, obesity and glucose tolerance as independent variables, were used to study their influence on phenotypic variables.

A multiple linear regression model with stepwise (probability of F to enter≤0.05, probability of F to remove≥0.10) introduction of age, BMI, waist-to-hip ratio, presence or absence of PCOS, serum C-reactive protein, ferritin and free testosterone concentrations, insulin sensitivity index and *Hp* genotypes (coded as *Hp^1^*/*Hp^1^* = 0, *Hp^2^/Hp^1^* = 1 and *Hp^2^/Hp^2^* = 2) as independent variables, was used to evaluate the contribution of these variables to the variability in serum haptoglobin concentrations. Analyses were performed using SPSS10 for the Macintosh (SPSS Inc, Chicago, Illinois). *P*<0.05 was considered statistically significant.

## Results

### Comparison of clinical, biochemical and metabolic variables among patients and controls

As expected by design the patients with PCOS and the non-hyperandrogenic controls showed no statistically significant differences in the mean BMI or in the distribution according to the weight classification (Table 1), yet because PCOS patients were younger than controls (25.4±6.3 *vs* 31.7±7.7 yr respectively, *P* = 0.001), age was introduced as a covariate in the following analyses.

**Table 1:** Distribution of PCOS patients and controls according to their weight classification.

		Weight classification *	
		Non-obese women (n = 127)	Obese women (n = 118)
PCOS (n = 141)	n (%)	79 (56%)	62 (44%)
	BMI (kg/m^2^, mean±SD)	24.4±3.6	37.0±5.4
Controls (n = 102)	n (%)	47 (46%)	55 (54%)
	BMI (kg/m^2^, mean±SD)	24.6±4.2	36.9±5.4

* Women were classified into obese (BMI≥30 kg/m^2^) or non-obese (BMI<30.0 kg/m^2^) subgroups according to their BMI.

No differences were observed between PCOS patients and controls in the distribution according to their weight classification (χ^2^ = 2.347, *P* = 0.126), or in their BMI (F = 1.580, *P* = 0.210).

PCOS patients presented with increased hirsutism scores, waist-to-hip ratio, fasting insulin and triglycerides, serum concentrations of total and free testosterone, androstenedione, 17-hydroxyprogesterone, dehydroepiandrosterone-sulfate and luteinizing-hormone (Table 2). On the contrary, the insulin sensitivity index as well as serum sex hormone-binding globulin and estradiol levels were reduced in the patients. Furthermore, there were no differences in the frequency of smoking or of abnormal glucose tolerance.

**Table 2:** Clinical and biochemical characteristics of PCOS patients and non-hyperandrogenic control women.

	PCOS patients (n = 141)			Controls (n = 102)			*P* value
Body mass index (kg/m^2^)	30	±	8	31	±	8	0.483
Smokers (n, %)	44		32	34		33	0.843
Waist-to-hip ratio	0.784	±	0.081	0.784	±	0.078	0.015
Hirsutism score	10.0	±	6.1	1.5	±	1.7	0.001
Haptoglobin (g/l)	1.4	±	0.5	1.5	±	0.5	0.990
C-reactive protein (mg/l)	3.4	±	4.2	3.7	±	4.6	0.639
Ferritin (pmol/l)	121	±	101	79	±	46	0.001
Total testosterone (nmol/l)	2.2	±	0.9	1.4	±	0.5	0.001
Free testosterone (pmol/l)	45	±	23	22	±	9	0.001
Sex hormone-binding globulin (nmol/l)	32	±	17	45	±	23	0.001
17-hydroxyprogesterone (nmol/l)	2.9	±	1.6	1.9	±	1.1	0.001
Androstenedione (nmol/l)	13.3	±	5.0	8.3	±	3.0	0.001
Dehydroepiandrosterone-sulfate (µmol/l)	6.0	±	2.5	4.2	±	2.1	0.001
Luteinizing hormone (IU/l)	6.5	±	3.9	5.1	±	3.1	0.040
Follicle-stimulating hormone (IU/l)	6.0	±	4.4	6.1	±	4.3	0.487
Estradiol (pmol/l)	154	±	108	236	±	215	0.002
Cholesterol (mmol/l)	4.4	±	0.9	4.7	±	1.0	0.705
High-density lipoprotein cholesterol (mmol/l)	1.3	±	0.5	1.3	±	0.4	0.416
Low-density lipoprotein cholesterol (mmol/l)	2.6	±	0.8	2.9	±	0.8	0.511
Triglycerides (mmol/l)	1.1	±	0.9	1.0	±	0.5	0.002
Fasting glucose (mmol/l)	5.0	±	0.5	5.1	±	0.5	0.510
Fasting insulin (pmol/l)	101	±	73	71	±	47	0.001
Insulin sensitivity index	4.3	±	2.9	5.9	±	3.9	0.001
Normal glucose tolerance (n, %)	113		80	79		77	0.635
Abnormal glucose tolerance (n, %)	28		20	23		23	

Results are means±SD or raw numbers and percentages. Continuous variables were submitted to a general linear model in which age was introduced as a covariate to correct for the difference in age observed between patients and controls. Categorical variables were submitted to Fisher's exact test.

### Haptoglobin and PCOS

Serum haptoglobin concentrations were not different among PCOS patients and non-hyperandrogenic controls (Table 2). Furthermore, the frequency of women presenting with serum haptoglobin concentrations above the references values for their haptoglobin genotype was similar in both groups (controls 25.5% vs PCOS patients 26.1%, χ^2^ = 0.010, *P* = 0.921).

After confirming that the haptoglobin genotype was distributed according to Hardy-Weinberg equilibrium in both PCOS patients and controls (data not shown), we found that the *Hp^2^* allele was more frequent in the former (62% *vs* 52%, χ^2^ = 5.191, *P* = 0.023), explaining a borderline significant tendency towards a higher frequency of *Hp^2^/Hp^2^* genotype, and to a reduced frequency of the *Hp^1^/Hp^1^* genotype, in PCOS patients compared with the controls (Table 3).

**Table 3:** Distribution of haptoglobin genotypes in the presence or absence of PCOS, obesity, or disordered glucose tolerance.

	Haptoglobin genotype		
	*Hp^1^/Hp^1^* n (%)	*Hp^2^/Hp^1^* n (%)	*Hp^2^/Hp^2^* n (%)
Whole population (n = 243)	48 (20)	107 (44)	88 (36)
PCOS patients (n = 141)	22 (16)	61 (43)	58 (41)
Controls (n = 102)	26 (26)	46 (45)	30 (29)
Non-obese women (n = 126)	24 (19)	56 (44)	46 (37)
Obese women (n = 117)	24 (20)	51 (44)	42 (36)
Normal glucose tolerance (n = 192)	42 (22)	82 (43)	68 (35)
Abnormal glucose tolerance (n = 51)	6 (12)	25 (49)	20 (39)

There was a borderline significant tendency towards an increased frequency of *Hp^2^/Hp^2^* genotype, and a decreased frequency of the *Hp^1^/Hp^1^* genotype, in PCOS patients compared with non-hyperandrogenic controls (χ^2^ = 5.220, *P* = 0.074 overall; χ^2^ = 3.650, *P* = 0.056 for a dominant model; χ^2^ = 3.521, *P* = 0.061 for a recessive model).

There were no statistically significant differences in the distribution of haptoglobin genotypes among the women classified according to their weight classification (χ^2^ = 0.082, *P* = 0.960) or to their glucose tolerance (χ^2^ = 2.610, *P* = 0.271).

**Figure 1:**
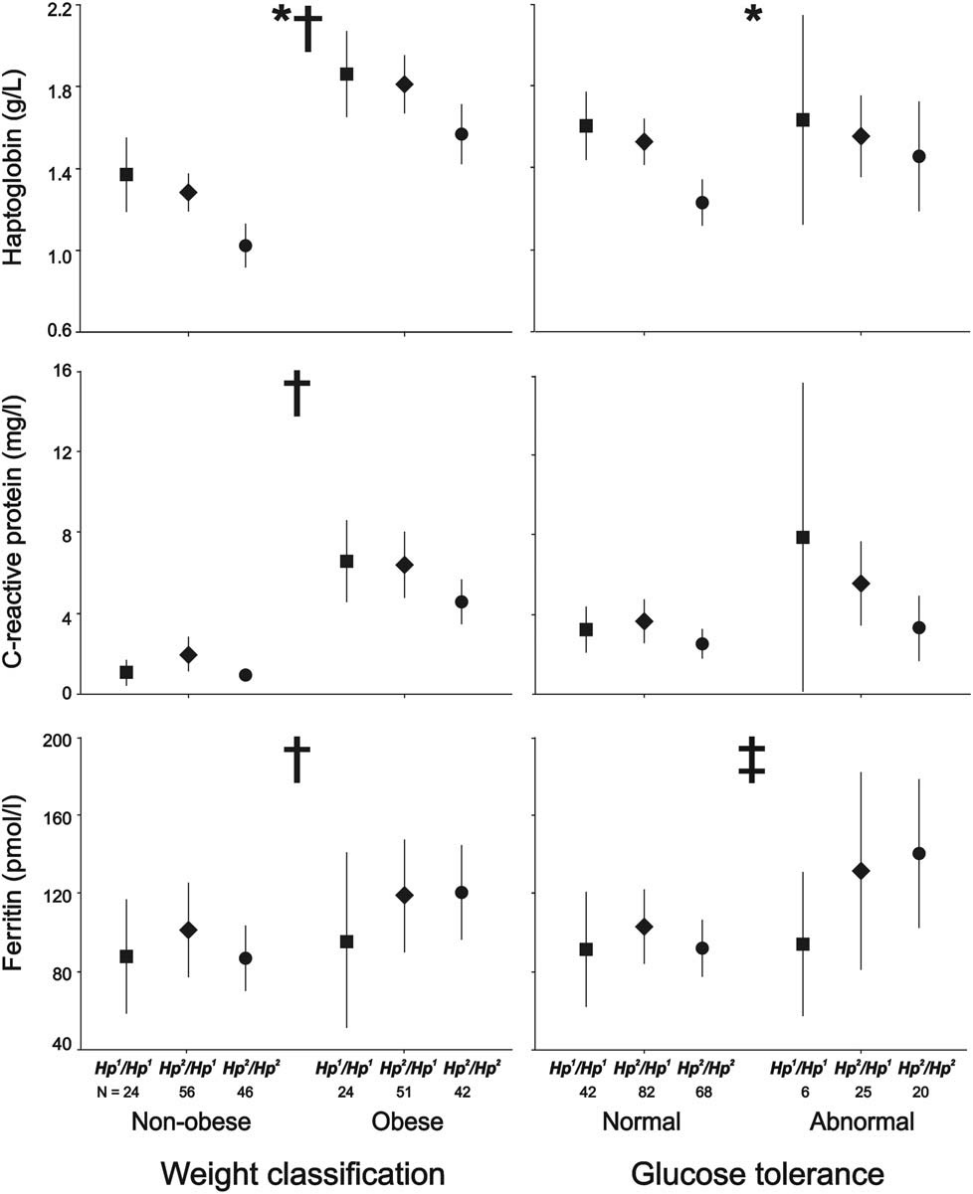
Serum haptoglobin, C-reactive protein and ferritin concentrations as a function of the haptoglobin genotype, obesity and glucose tolerance. Data are means and 95% confidence intervals. The numbers below the x-axis are the number of subjects in each subgroup. *P<0.05 or less between haptoglobin genotypes; †*P*<0.05 or less between obese and non-obese women. ‡*P*<0.05 or less between women with normal or abnormal glucose tolerance.

Finally, we confirmed that women with the *Hp^2^/Hp^2^* genotype had mildly reduced circulating haptoglobin concentrations compared with women presenting the *Hp^2^/Hp^1^* and *Hp^1^/Hp^1^* genotypes (Figure 1) yet the 10% difference in the frequencies of haptoglobin genotypes among patients and controls, although statistically significant, was not large enough to cause an actual difference in their mean serum haptoglobin concentrations (Table 2).

### Haptoglobin and obesity

Serum haptoglobin levels were increased in obese women irrespective of PCOS status (Figure 1). Accordingly, obese women presented with a 5-fold increase in the frequency of women presenting with elevated serum haptoglobin concentrations for their haptoglobin genotype (non-obese 8.7%, obese women 44.1%, χ^2^ = 39.73, *P*<0.001).

Of note, the increase in serum haptoglobin concentrations with obesity was unrelated to the haptoglobin polymorphism, because its genotypes were equally distributed among non-obese and obese women (Table 3).

### Haptoglobin and abnormalities of glucose tolerance

Serum haptoglobin concentrations were not different when women were classified according to their normal or abnormal glucose tolerance (Figure 1), and the frequency of subjects presenting with increased serum haptoglobin levels for their genotype were not different depending on their glucose tolerance (normal glucose tolerance 24.0%, abnormal glucose tolerance 33.3%, χ^2^ = 1.844 *P* = 0.174). Finally, haptoglobin genotypes were similarly distributed among the groups with normal or abnormal glucose tolerance (Table 3).

### Impact of the haptoglobin polymorphism, PCOS, obesity and abnormalities of glucose tolerance on serum ferritin and C-reactive protein concentrations

The haptoglobin genotype did not influence serum ferritin and C-reactive protein concentrations (Figure 1). Serum ferritin concentrations were higher in PCOS patients when compared with non-hyperandrogenic controls (Table 2), were also higher in obese women when compared with non-obese women (Figure 1), and increased in the women presenting with abnormal glucose tolerance (Figure 1). On the contrary, serum C-reactive protein concentrations increased in obese women compared to their non-obese counterparts and tended to increase (*P* = 0.079) in the women presenting with abnormal glucose tolerance (Figure 1), but were similar in PCOS and non-hyperandrogenic women (Table 2).

### Determinants of serum haptoglobin levels in premenopausal women

A multiple regression model (R^2^ = 0.498, F = 58.6, *P*<0.0001) demonstrated that 50% of the variation in serum haptoglobin concentrations was explained by the variation in serum C-reactive protein concentrations (β = 0.405, *P*<0.001), BMI (β = 0.251, *P*<0.001), *Hp* genotypes (β = −0.207, *P*<0.001) and insulin sensitivity index (β = −0.167, *P* = 0.001). Age, PCOS, waist-to-hip ratio, ferritin and free testosterone concentrations were excluded by the stepwise regression model.

## Discussion

To our best knowledge, we here present the first data regarding serum haptoglobin concentrations and its genotypes in patients with PCOS. The most important finding of our present study is that, compared with non-hyperandrogenic women, PCOS patients presented with an increased frequency of the *Hp^2^* allele (and a borderline significant tendency towards an increased frequency of the *Hp^2^/Hp^2^* genotype).

The importance of the association of PCOS with *Hp^2^* alleles is that this polymorphism influences the functional properties of haptoglobin in a way that might contribute to the known clustering of cardiovascular risk factors in PCOS patients [10] even in the absence of actual differences in serum haptoglobin levels.

On the one hand, the haptoglobin polymorphism causes a gene-dosage effect resulting in a graded decrease in the haptoglobin ability to bind hemoglobin, in the antioxidant capacity and in prostaglandin inhibition, with the *Hp^2^/Hp^2^* genotype expressing the weakest protein activities [14]. On the other hand, the haptoglobin polymorphism also results in a graded increase in the angiogenic and inflammatory effects of haptoglobin, with the *Hp^2^/Hp^2^* genotype expressing the strongest properties [14]. Therefore, the increased frequency of *Hp^2^* alleles in our PCOS patients might also play a role in the previously reported low-grade chronic inflammation [28] and increased oxidative stress [9] of these women, even when serum haptoglobin concentrations were not altered in these PCOS women.

Supporting a role for the haptoglobin polymorphism in the development of atherosclerotic disease and cardiovascular events, the Hp 2-2 phenotype increases the risk for micro- and macrovascular complications in diabetic patients [29] possibly because this phenotype facilitates the generation of reactive oxygen species, and oxidative stress is involved in myocardial damage and atherosclerosis. Furthermore, in the ApoE knock-out murine model of atherosclerosis, *Hp^2^/Hp^2^* mice obtained by targeted insertion of a murine type 2 *Hp* allele into the *Hp* locus by homologous recombination show increased iron deposition, lipid peroxidation and macrophage accumulation within atherosclerotic plaque, increasing the risk of rupture and thrombosis [30]. Interestingly, PCOS patients show early markers of atherosclerosis such as increased carotid intima-media thickness and coronary artery calcification from young ages [31], [32].

Of note, the difference in the frequency of haptoglobin genotypes was actually caused by a relatively low frequency of *Hp^2^* alleles in the controls when compared to that found in the general population of other countries whereas, on the contrary, the frequency found in the patients was similar to that reported in other Caucasians populations.

This surprising finding has two possible explanations: first, the frequencies of haptoglobin alleles in the Spanish population has not been established, and may be lower compared with those found in other European controls. Second, our controls were selected as to have no known metabolic or hyperandrogenic disorder, and considering the high prevalences of these conditions in the general population, this selection bias may explain a reduced frequency of *Hp^2^* alleles in them. Nevertheless, the distribution of *Hp* alleles was in Hardy-Weimberg equilibrium in both PCOS patients and in controls, ruling out significant population stratification in our series.

The α chain haptoglobin polymorphism influences circulating haptoglobin concentrations to a lesser degree compared with its impact on the functionality of the protein [14]. This may explain why PCOS patients, despite carrying *Hp^2^* alleles more frequently than controls, did not have reduced serum haptoglobin levels. Also, the lowering effect on serum haptoglobin levels of carrying *Hp^2^* alleles may have been obscured in the PCOS patients presented here because these women were insulin resistant compared with the controls, and insulin resistance actually increases serum haptoglobin levels [33] as our data also demonstrate.

Our present results do not suggest that the haptoglobin polymorphism influences the increased serum ferritin concentrations of PCOS patients, in sharp contrast with the findings in male hemochromatosis patients, in whom the Hp 2-2 phenotype increases iron overload and the expression of the hemochromatosis phenotype [17]. Although the ferritin lowering effect of regular menstrual iron losses [34] might contribute to explain this discrepancy, most of the PCOS patients had chronic oligomenorrhea, and our previous studies on PCOS patients suggest that insulin resistance, and not genetic factors, is one of the major determinants of increased serum ferritin concentrations and iron stores in these women [12]. Similarly, the haptoglobin polymorphism did not influence serum C-reactive protein concentrations in our population, yet the possible effect of the polymorphism on local inflammatory mechanisms may have been obscured by the strongest influence of obesity on the serum levels of this inflammatory marker [35].

The impact of weight excess on serum haptoglobin concentrations and the other circulating inflammatory markers studied here merits further comment. Our present results indicate that serum haptoglobin concentration, and the number of women presenting with serum levels above the upper limit of the normal range, increase with obesity. These results confirm the previously reported association of circulating haptoglobin concentrations with BMI [18], [36], which might be dependent on the expression of haptoglobin in adipose tissue [18]. Furthermore, obesity also increases serum C-reactive protein and ferritin concentrations pointing to the existence of chronic inflammation and increased body iron stores in obesity and other disorders characterized by the presence of insulin resistance.

Even when serum haptoglobin levels increase with insulin resistance and obesity [18], [33], [36], neither serum haptoglobin levels nor the haptoglobin genotype were related to the development of disorders of glucose tolerance in our series of young premenopausal women. Therefore, haptoglobin does not appear to be involved in the failure of compensatory endogenous hyperinsulinism to maintain glycemia within the normal range, which is the hallmark of abnormal glucose tolerance in insulin resistant subjects. On the contrary, increased body iron stores may have contributed to this failure [13], because serum ferritin levels were clearly increased in the women who had abnormal glucose tolerance in our series. Of note, chronic inflammation might play a similar role, yet the tendency towards an increase in serum C-reactive protein levels in women presenting with abnormal glucose tolerance did not reach statistical significance.

Our study was limited, however, by a relatively low statistical power for detection of differences between the small subgroups resulting from categorization by presence or absence of metabolic traits. This limitation was especially evident when considering glucose tolerance, and therefore a possible influence of haptoglobin levels or genotypes on the development of disorders of glucose tolerance cannot be definitely excluded by our present results.

Finally, the results of the multiple regression model indicates that serum haptoglobin concentrations are determined in premenopausal women by several factors including BMI, serum C-reactive protein concentrations and the insulin sensitivity index, in addition to haptoglobin genotypes. Overall, the result of the regression analysis suggests that adipose tissue, and also the chronic inflammatory milieu (possibly reflecting the action of inflammatory cytokines such as TNF-α and IL-6 on the liver) and the insulin resistant state associated with obesity, up-regulate haptoglobin secretion contributing to its circulating concentrations. On the contrary, the haptoglobin *Hp^2^/Hp^2^* genotype resulted in a mild decrease in serum haptoglobin concentrations in our premenopausal women, as has been shown in other populations [14].

In summary, the association of PCOS with *Hp^2^* alleles of haptoglobin suggests that the functionality of this protein might be altered in these women, even when its circulating concentrations are not altered because their insulin resistance counteracts the decrease in circulating haptoglobin usually associated with *Hp^2^* alleles. Also, our present results indicate that serum haptoglobin levels of young premenopausal women are mostly dependent on the haptoglobin polymorphism and on obesity, with insulin resistance and chronic inflammation possibly contributing to this association.
